# Genome Sequence of a Thermostable Avirulent Newcastle Disease Virus Isolated from Domestic Ducks in China

**DOI:** 10.1128/MRA.01218-19

**Published:** 2019-11-21

**Authors:** Li Li, Min Wang, Guokang Wang, Lintao Li, Hongling Wang, Qingping Luo, Yu Shang, Tengfei Zhang, Huabin Shao, Guoyuan Wen

**Affiliations:** a Institute of Animal Husbandry and Veterinary Sciences, Hubei Academy of Agricultural Sciences, Wuhan, China; b Key Laboratory of Prevention and Control Agents for Animal Bacteriosis (Ministry of Agriculture), Wuhan, China; c Hubei Key Laboratory of Animal Embryo and Molecular Breeding, Wuhan, China; DOE Joint Genome Institute

## Abstract

Newcastle disease virus strain D4 was isolated from healthy ducks in Hubei, China. The D4 isolate has a genome length of 15,186 nucleotides and is classified as genotype I of class II. Thermostability and pathogenicity tests demonstrate that D4 is a thermostable avirulent strain.

## ANNOUNCEMENT

Newcastle disease (ND) is a highly infectious and often fatal avian disease caused by virulent strains of Newcastle disease virus (NDV), which belongs to the genus *Avaluvirus* within the family *Paramyxoviridae*. ND is a worldwide threat and is endemic in many developing countries (1). Control of ND by vaccination is a common practice in many countries worldwide. Most of the vaccine strains are sensitive to heat, such as LaSota and B1, and subsequently require a cold chain to maintain their quality during transport and storage. A few vaccine strains are thermostable, such as V4 and I_2_ (2, 3). The viral titer of the V4 vaccine decreased from 10^10.4^ to 10^9.3^ 50% egg infective dose (EID_50_) after 14 days of storage at 27 to 32°C (4). The thermostable vaccines have been used widely to protect village chickens against ND (5, 6). However, most of the thermostable vaccine strains are derived from chickens, and the thermostable avirulent NDVs from other species and their corresponding genomic sequences have not been reported.

A set of 21 cloacal swab samples were collected from healthy ducks on a farm in Hubei, China, in 2016 and were individually inoculated into 10-day-old specific-pathogen-free chicken embryos. NDV strain D4 was isolated from a cloacal swab sample by means of a hemagglutinin-inhibiting assay (7). The infective titer of strain D4 in chicken embryos was 10^9.6^ EID_50_/ml. The D4 virus was proven to be an avirulent strain with an intracerebral pathogenicity index value of 0.63 and a mean death time at a minimal lethal dose value of 111.2 h (7). The thermostability of the D4 virus at 56°C was examined *in vitro* by performing the hemagglutinin (HA) and infectivity thermostability test, as previously described (8). The mean times for a 90% decrease in HA activity and infectivity of D4 were 82.5 min and 6.7 min, respectively. According to the criteria for NDV thermostability (9), the NDV isolate D4 is a thermostable virus.

**FIG 1:**
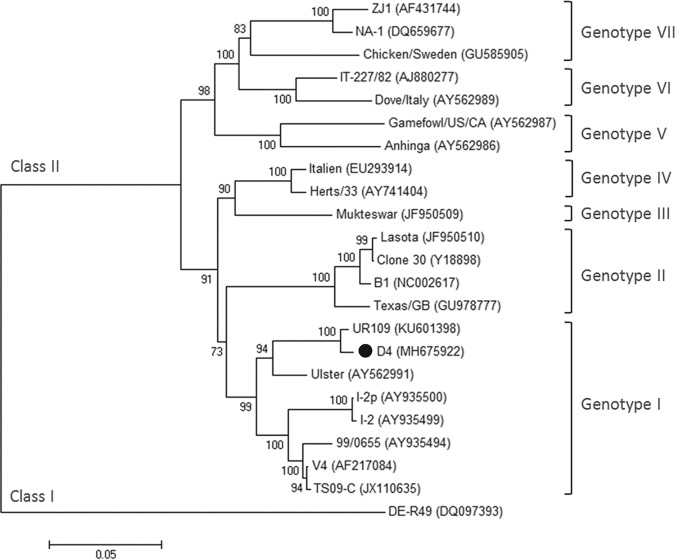
Phylogenetic analysis of NDV isolates based on the complete F gene sequences. The F gene sequences of 22 NDV isolates were obtained from the NCBI GenBank database and aligned with that of strain D4 using Clustal X 1.83. The phylogenetic tree was constructed by using the neighbor-joining algorithm in MEGA 4.0 with 1,000 bootstrap replicates to assign confidence to groupings. The F gene sequence of thermostable strain D4 determined in this study is marked with a black circle. The GenBank accession numbers of the NDV isolates are shown in parentheses. The genotypes of the NDV strains are indicated at the right side of the diagram.

For the complete genome sequencing, viral RNA was extracted from allantoic fluid infected with strain D4 using TRIzol (Invitrogen, USA). The 10 genomic fragments covering the whole genome of strain D4 were reverse transcriptase PCR (RT-PCR) amplified using 10 primer sets (10). The RT-PCR products were purified and sequenced using Sanger dideoxy sequencing technology at least three times to ensure the consensus sequence. The 3′ leader and 5′ trailer sequences of strain D4 were determined using 3′ rapid amplification of cDNA ends (3′ RACE) and 5′ RACE, respectively (11). The sequences were compiled and assembled using SeqMan (Lasergene). The assembled genomic sequence of NDV strain D4 was 15,186 nucleotides long, with a 47.31% GC content. A preliminary BLAST search of the complete genome sequence in the NCBI nonredundant/nucleotide (nr/nt) database (http://blast.ncbi.nlm.nih.gov) showed 98.8% nucleotide identity with NDV strain UR109 derived from Uria aalge. The alignment of amino acid sequences and the estimation of amino acid identities were performed using the Clustal W method in the MegAlign program (Lasergene). The amino acid identities of the NP, P, M, F, HN, and L proteins between D4 and UR109 were 99.8, 98.7, 99.7, 99.5, 99.3, and 99.6%, respectively. However, sequence alignment showed that the D4 virus had a 36-amino-acid deletion at the C terminus of the HN protein, which was a distinctive difference from the UR109 strain. The phylogenetic analysis based on the complete F gene using the MEGA 4.0 software demonstrated that NDV strain D4 is a member of genotype I, together with the UR109 and Ulster isolates (Fig. 1).

We report the identification and genome sequence of the thermostable avirulent NDV strain D4 isolated from ducks in China. It will help us better understand the epidemiology of NDV in waterfowl and develop a thermostable ND vaccine for ducks.

### Data availability.

The complete genome sequence of NDV strain D4 has been deposited in GenBank under the accession no. MH675922.
